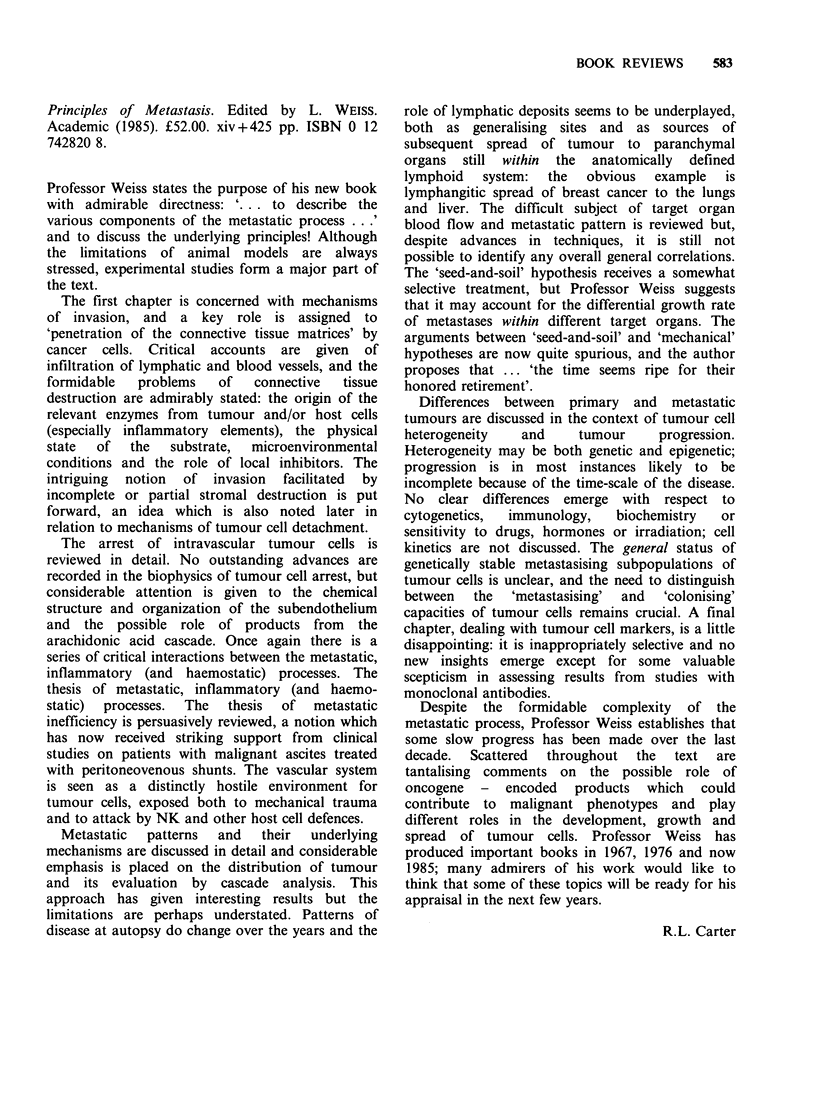# Principles of Metastasis

**Published:** 1986-04

**Authors:** R.L. Carter


*Principles of Metastasis.* Edited by L. WEISS. Academic (1985). £52.00. xiv+425 pp. ISBN 0 12 742820 8.

Professor Weiss states the purpose of his new book with admirable directness: '. . . to describe the various components of the metastatic process . . .' and to discuss the underlying principles! Although the limitations of animal models are always stressed, experimental studies form a major part of the text.

The first chapter is concerned with mechanisms of invasion, and a key role is assigned to 'penetration of the connective tissue matrices' by cancer cells. Critical accounts are given of infiltration of lymphatic and blood vessels, and the formidable problems of connective tissue destruction are admirably stated: the origin of the relevant enzymes from tumour and/or host cells (especially inflammatory elements), the physical state of the substrate, microenvironmental conditions and the role of local inhibitors. The intriguing notion of invasion facilitated by incomplete or partial stromal destruction is put forward, an idea which is also noted later in relation to mechanisms of tumour cell detachment.

The arrest of intravascular tumour cells is reviewed in detail. No outstanding advances are recorded in the biophysics of tumour cell arrest, but considerable attention is given to the chemical structure and organization of the subendothelium and the possible role of products from the arachidonic acid cascade. Once again there is a series of critical interactions between the metastatic, inflammatory (and haemostatic) processes. The thesis of metastatic, inflammatory (and haemostatic) processes. The thesis of metastatic inefficiency is persuasively reviewed, a notion which has now received striking support from clinical studies on patients with malignant ascites treated with peritoneovenous shunts. The vascular system is seen as a distinctly hostile environment for tumour cells, exposed both to mechanical trauma and to attack by NK and other host cell defences.

Metastatic patterns and their underlying mechanisms are discussed in detail and considerable emphasis is placed on the distribution of tumour and its evaluation by cascade analysis. This approach has given interesting results but the limitations are perhaps understated. Patterns of disease at autopsy do change over the years and the role of lymphatic deposits seems to be underplayed, both as generalising sites and as sources of subsequent spread of tumour to paranchymal organs still *within* the anatomically defined lymphoid system: the obvious example is lymphangitic spread of breast cancer to the lungs and liver. The difficult subject of target organ blood flow and metastatic pattern is reviewed but, despite advances in techniques, it is still not possible to identify any overall general correlations. The 'seed-and-soil' hypothesis receives a somewhat selective treatment, but Professor Weiss suggests that it may account for the differential growth rate of metastases *within* different target organs. The arguments between 'seed-and-soil' and 'mechanical' hypotheses are now quite spurious, and the author proposes that ... 'the time seems ripe for their honored retirement'.

Differences between primary and metastatic tumours are discussed in the context of tumour cell heterogeneity and tumour progression. Heterogeneity may be both genetic and epigenetic; progression is in most instances likely to be incomplete because of the time-scale of the disease. No clear differences emerge with respect to cytogenetics, immunology, biochemistry or sensitivity to drugs, hormones or irradiation; cell kinetics are not discussed. The *general* status of genetically stable metastasising subpopulations of tumour cells is unclear, and the need to distinguish between the 'metastasising' and 'colonising' capacities of tumour cells remains crucial. A final chapter, dealing with tumour cell markers, is a little disappointing: it is inappropriately selective and no new insights emerge except for some valuable scepticism in assessing results from studies with monoclonal antibodies.

Despite the formidable complexity of the metastatic process, Professor Weiss establishes that some slow progress has been made over the last decade. Scattered throughout the text are tantalising comments on the possible role of oncogene – encoded products which could contribute to malignant phenotypes and play different roles in the development, growth and spread of tumour cells. Professor Weiss has produced important books in 1967, 1976 and now 1985; many admirers of his work would like to think that some of these topics will be ready for his appraisal in the next few years.

R.L. Carter